# Carer involvement in the assessment of personal recovery: A naturalistic study of assertive community treatment in Norway

**DOI:** 10.3389/fpsyt.2023.1135135

**Published:** 2023-03-27

**Authors:** Pravin Israel, Torleif Ruud, Bente Weimand

**Affiliations:** ^1^Faculty of Social Sciences, University of Stavanger, Stavanger, Norway; ^2^Faculty of Health Studies, VID Specialized University, Oslo, Norway; ^3^Institute of Clinical Medicine, Faculty of Medicine, University of Oslo, Oslo, Norway; ^4^Division of Mental Health, Department of Special Psychiatry, Akershus University Hospital, Lørenskog, Norway; ^5^Department of Health, Social, and Welfare Studies, University of South-Eastern Norway, Kongsberg, Norway

**Keywords:** personal recovery, recovery, family carers, caregivers, serious mental illness, psychosis, multi-informant method, assertive community treatment (ACT)

## Abstract

**Background:**

The user and carer movements have come a long way in becoming embedded in mainstream mental health services for individuals with serious mental illness. However, implementing recovery-oriented practice continues to be plagued by an individualistic clinical focus. The carers do not feel integrated despite policies and best intentions. The implementation of Assertive Community Treatment (ACT) provided an opportunity to involve the carers and compare their assessment of personal recovery with the users.

**Aims:**

The aims of this study were to examine (i) how family carers and users differed in their assessment of personal recovery, (ii) whether familial and personal relationships influenced how carers assess personal recovery of users, and (iii) if the experience of family carers with the ACT team was associated with personal recovery.

**Methods:**

The naturalistic, explorative study recruited 69 users and 36 family carers from 12 Norwegian ACT teams. The users and carers assessed the user's personal recovery. Family carers also reported their experience and satisfaction with the ACT teams. Analyses included independent and paired sample T-tests and correlation analysis.

**Results:**

Family carers were significantly more conservative than the users' assessment of the intrapsychic and interpersonal subscales of personal recovery. The pattern held true whether the family carers were matched to the users or part of the total sample. Lastly, there was a significant negative correlation between the family carer's experience of cooperation with the ACT team and their assessment of the user's intrapersonal process of recovery.

**Conclusions:**

The results of our study were consistent with previous research on carer involvement in MHS. However, it is the first study that engaged carers and assessed personal recovery of the users of ACT services. Discrepancy between carers and users is the rule. Clinicians are encouraged to embrace the discrepancy and diversity carers bring and learn the methodology of multi-informant assessments. There also is a need to address, update, and integrate the personal, familial, and relational aspects of recovery. Modification of recovery measures such as QPR and their creative use with carers has the potential to generate valuable third-party information and to involve them meaningfully in mental health services.

## Introduction

User and family-driven carer movements grew out of dissatisfaction with mainstream mental health services (MHS) and its perceived short-sighted and paternalistic attitude (1, 2). Consequently, it raised awareness that service users (henceforth known as users) and family carers (henceforth known as carers) are key stakeholders in the design and delivery of contemporary MHS for individuals with serious mental illness (SMI). Their subsequent recruitment to influential posts on boards and committees of service organizations provided them with a strong voice and presence (3). Further, in recognition of the enormous contribution of carers and the risks they face, government policies and guidelines were promulgated to strengthen their position and participation. However, there is still discontent among carers who feel that they provide vital care and support to users, but feel excluded by MHS (4, 5). In Norway, the association for carers feel that there is a gap between the need of carers for support and what they receive.

The user movement has had help from, among others, the recovery movement. Traditionally, recovery was defined by trained professionals from a medical model that characterized mental illness (6). Subsequently, clinical recovery was defined as the reduction or remission of symptoms and the restoration of functional levels. The recovery movement was characterized by internal, subjective, and lived experiences of pursuing a meaningful and satisfying life despite the limitations imposed by severe mental illness (7–9). Consequently, recovery-based services focused on processes where users actively navigated unique and multiple pathways, facilitated by mental health professionals and peers (3). On the research front, the conceptual framework of recovery- CHIME defined personal recovery as a synthesis of connectedness, hope, identity, meaning in life, and empowerment, which provided a platform to launch future research and practice (10, 11).

The carer movement has not yet reached the level of success as the user movement. From a service provider's perspective, involving carers is complicated given multiple barriers, such as the lack of consent from users, confidentiality, attitudes and beliefs of carers regarding specific clinical interventions, and individualistic focus of the mental health care system (12, 13). Further, discrepancies in the information provided by carers and users are difficult for clinicians to interpret and apply to concrete clinical situations and plans. The literature on multiple informants in clinical services shows that the discrepancy between informants is the rule rather than the exception (14–16). On the clinical side, more two decades ago, Brown & Rutter (17) defined Expressed Emotion as the emotional environment in which the patient resides, as exhibited by the manner in which their relatives communicate about them. Studies around expressed emotions enriched our understanding of family dynamics that may be associated with the mental illness (18). Many studies documented the enduring reality of the carers' high burden of care on, among others, their physical and mental health, social isolation, and negative impact on their quality of life (19). Nevertheless, there are also studies that demonstrated positive effects of carer involvement, such as treatment adherence (20), treatment outcomes (21), fewer hospital admissions and shorter inpatient stay (22), and effect sizes for family interventions are comparable to medications (23). Several family-based interventions were developed. For example, two clinical trials in family-assisted assertive community treatment demonstrated positive outcomes for patients with schizophrenia (24, 25). A recent feasibility study aimed at systematically including carers (*n* = 30) reported improved communication between the users and carers, and the clinicians reported that the procedure was simple, straightforward, and helpful (26). However, many family-centered clinical interventions are time limited and do not meet the scope of mental health rehabilitation needs of people with SMI.

Previous studies show that carers have been involved in several ways. For example, a large clinical trial that evaluated the effectiveness and safety of the antipsychotic medication showed that systematic involvement of carers was associated with increased adherence to treatment and reduced need for rehospitalization (21). Furthermore, psycho-education programs were an important tool to prepare and train carers to provide, among others, support to the user outside the MHS context. Studies involving people with serious mental illnesses such as schizophrenia and bipolar disorder documented favorable results using the family psychoeducation model (27, 28). A recent feasibility study aimed at systematically including carers (*n* = 30) reported improved communication between the users and carers, and the clinicians reported that the procedure was simple, straightforward, and helpful (26). At the organizational level factors such as satisfaction with MHS are also known to have an impact on user outcomes. In fact, the user satisfaction model was built on the notion that the perception of being included and cared for facilitated positive care processes and produced favorable treatment outcomes. Subsequent studies corroborated these assumptions, and user satisfaction came to be adopted as a proxy for quality of care (27, 29). Although carers are not direct beneficiaries of MHS, their feeling of satisfaction with the services could play an important role, especially when users are indisposed due to episodes that render them incapable of making informed decisions. The carer's beliefs regarding the quality of services and expectations would influence the level of support and motivation they provide to the user.

Current practices in MHS are the foundation for the seeding and development of new ideologies, innovations, and models of care. However, inconsistencies and tensions arise as a natural part of reabsorbing, accommodating, and implementing these innovations in MHS. For instance, while government policies and guidelines mandate the inclusion of carers, there continues to be a lack of knowledge and training about the meaningful involvement of carers in the treatment and rehabilitation of users with SMI (3). Innovative and newer ideologies do not always come with explicit instructions, among others, on how to integrate it with ACT services or how to involve carers. Further, against the backdrop of an individualistic recovery agenda, researchers have noted recovery-oriented services overshadow interpersonal, relational, and social aspects of recovery, and by extension, carer involvement (30–32). Better integration of carers into recovery-oriented services needs to address the current understanding of recovery at the conceptual, ideological level (1, 33), and practical and operational level (34–36). For instance, the concept of personal recovery has been criticized for being egocentric, placing undue responsibility on the individual, and diminishing the role of a strong supportive network to successfully navigate the recovery process (1, 36). There are voices who wonder if personal recovery can be conceptualized in any other way than interpersonal, relational, and social (32, 35, 37).

The context for this study is the national implementation in Norway of the well-known and documented program called the Assertive Community Treatment (ACT). ACT is a highly individualized approach to the community-based care of individuals with severe and persistent mental illness (38) to support a life outside psychiatric institutions (39–41). Multidisciplinary teams of mental health professionals provide a comprehensive range of services, including medication management, case management, crisis intervention, and psychosocial rehabilitation, which are tailored to the specific needs of each individual. The overarching goal of ACT is to provide individuals with the support they need to manage their mental illness, live independently in the community, and achieve their personal goals. Historically, the ACT teams targeted deinstitutionalized persons living alone or in group homes and took care of the whole range of needs. However, since carer involvement is mandated by the Norwegian authorities and all mental health services are bound by health legislation and clinical guidelines (40–42), we expected carers to report satisfactory participation in ACT services for users. However, the carers of ACT users in Norway reported that while the new services were marginally better than the previous mainstream MHS, more than half did not feel included (43). Further, since recovery orientation has come to be an essential part of MHS, assessment of personal recovery is an important agenda. However, based on the CHIME study, an evaluation of MHS in Europe, Asia, Australia, and USA was undertaken, and the results showed recovery to be the least integrated measure in MHS (44). Given the non-existence of clinical studies showing carer involvement in services provided by ACT teams, this study sought to involve carers and provide them an opportunity to contribute by assessing, among others, personal recovery of the ACT service users.

## Aims of the study

The overall aim of this study was to explore the clinical characteristics and possibilities of including carers' assessment of personal recovery among Norwegian ACT users. Specific research questions were (i) How do carers and users differ in assessing the user's personal recovery? (ii) Does the familial, personal, and long-standing familiarity of users and carers influence their appraisal of personal recovery? (iii) Is there an associated between the family carer's experience of being included by the ACT team and their assessment of personal recovery?

## Methods

### Design and participants

**Figure 1:**
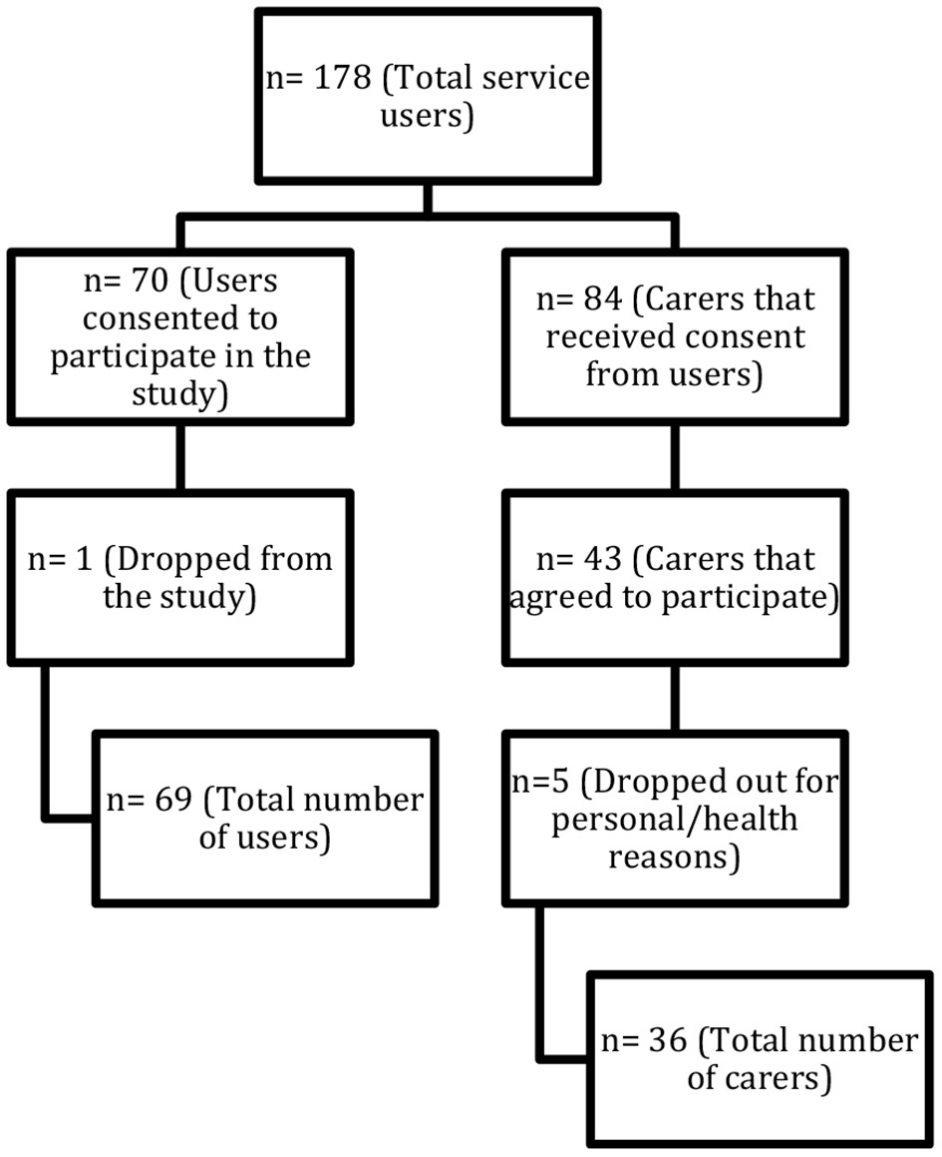
Flowchart showing recruitment of participants for the study.

The study was carried out as a naturalistic and exploratory study. Participants were recruited from the national implementation of the ACT model in Norway. Figure 1 shows the recruitment pathway of the 178 service users recruited to 12 ACT teams and their carers. Seventy service users gave their consent to participate in the national evaluation, and one user dropped out of the study. At the same time that users consented to participate, they were also asked to point out a carer and consent to their participation in the current study. The average age of the users was 40 years, and 32 % were women, and the average age of the carers was 60 years, and 78 % were women. Carers in this study were close family members. There were 26 (72 %) parents and 7 (19 %) siblings. There was also one foster parent, one spouse/partner, and one child over the age of 18 years.

### Measures

The interview consisted of closed-ended items from the Questionnaire about the Process of Recovery- QPR (40, 41). The QPR consists of 22 items with a 5-point Likert scale. In our analyses, we used 0 to mean "completely disagree” and 4 to mean “completely agree”. The QPR consists of two subscales, the intrapsychic process and the interpersonal process. The instrument had good internal consistency for the intrapsychic process subscale (a = 0.94) and the interpersonal subscale (a = 0.77). The carers reported on a modified QPR, where the sentence structure was modified to third person to elicit the recovery of the user. For example, the item “I am able to develop positive relationships with other people” was modified to “the patient is able to develop positive relationships with other people.” Cronbach's alpha values for the modified QPR from our study were 0.93 for the intrapersonal process subscale and 0.84 for the interpersonal process scale. Furthermore, we collected data from carers using the Family Involvement and Alienation Questionnaire-FIAQ (42). The instrument was developed in Sweden and consists of two scales, the experience of approach (a = 0.97) and the feeling of alienation (a = 0.89). We used the experience of approach scale in our study, which consisted of three subscales, openness (a = 0.89), confirmation (a = 0.91), and cooperation (a = 0.90).

### Data collection

Data was collected from the carers and the users by two independent research and co-research groups which used similar procedures. Data collection from the user group is described by Lofthus et al. (43). In short, a research group developed a questionnaire consisting of, among others, established instruments with acceptable psychometric properties. Co-researchers with lived experience as users of MHS (*n* = 9) conducted structured interviews with the ACT users after receiving appropriate training. Concordantly, the data collection from the carer group was collected by another research group which followed the same procedure for developing the questionnaire, recruiting co-researchers, and data collection. The co-researchers (*n* = 8) in the family carer group were carers of individuals with SMI and members of the National Association of Families of Mental Health (LPP in Norwegian). Their training consisted of a two-day didactic seminar that included a presentation of the ACT project, a scientific method for evaluating mental health, and dry runs of the questionnaire and structured interview process. There was a particular focus on biases and dilemmas in conducting research interviews. Additionally, the research group provided ongoing supervision during data collection.

### Statistical analyses

We conducted three analyses to address the research questions. Two analyses were based on the data available from the total groups of carers (*n* = 36) and users (*n* = 69). We used independent group *T*-tests and effect size calculations to analyze the differences between the total groups. However, due to the naturalistic nature of the study and the difficulties in obtaining consent from users to recruit their caregivers, there were only 18 pairs of matched users and caregivers. Since the carers and users in the subgroup used the same assessment, we conducted paired sample *T*-tests to analyze the differences. Finally, we used Pearson's correlations to analyze the association between the carers (*n* = 36) experiences with the ACT teams and their assessment of personal recovery of the users.

### Ethical approval

The study was approved by the Southeast Regional Ethics Committee for Medical and Health Research (Reg. No. 2010/1196a) and the data protection officer at Akershus University Hospital (Reg. No 2012/094).

## Results

Independent between-group analyses presented in Table 1 show the mean scores and standard deviation of the two scales of QPR (intrapsychic process and interpersonal process). The mean scores of the carer group were at the midpoint for both subscales. Independent group *T*-tests confirmed that carers reported significantly lower than the users on both the subscales of intrapsychic process and interpersonal process. The estimation of Cohen's D showed a medium effect size between the groups for both subscales (*d* = 0.69 for the intrapsychic process and *d* = 0.77 for the interpersonal process).

**Table 1:** The mean scores and standard deviation of the two scales of Questionnaire of Personal Recovery (QPR) for users (*n* = 69) and carers (*n* = 36).

	**Informant**	***N***	**Mean**	**SD**	**Independent *T*-test**
QPR intrapsychic process	Users	69	2.70	0.60	*t*(103) = 2.34, *p = 0.0*2
	Carers	36	2.02	0.83	
QPR interpersonal process	Users	69	2.71	0.69	*t*(103) = 3.45, *p* < 0.001
	Carers	36	2.17	0.89	

We analyzed the pattern of assessment of personal recovery in the matched carer-user subgroup (*n* = 18). Paired sample *T*-test presented in Table 2 showed that close family reported significantly lower than the users both on the subscale of the intrapsychic process and on the subscale of the interpersonal process of personal recovery.

**Table 2:** The mean scores and standard deviation of QPR sub-scales for users and carers in the matched sub-group (*n* = 18).

	**Informants**	**N**	**Mean**	**SD**	**Paired sample *T*-test**
QPR: Intrapsychic process	Users	18	2.84	0.48	*t*(17) = 3.27, *p = 0.0*04)
	Carers	18	2.10	0.81	
QPR: Interpersonal process	Users	18	2.82	0.56	*t*(17) = 2.53, *p = 0.0*21)
	Carers	18	2.38	0.80	

Next, we assessed if the carers' experience and satisfaction with the ACT team were correlated with their evaluation of the users' personal recovery. Table 3 shows the correlation matrix between the Approach subscales of the FIA-Q and both subscales of the QPR. There was only one significant correlation between the various subscales of the carers' experience and satisfaction with the ACT team and their assessment of personal recovery of users. The intrapsychic process had a significant negative correlation with the experience of cooperation subscale.

**Table 3:** The correlation matrix between the subscales of FIA-Q and QPR reported by carers (*n* = 36).

	**Descriptive statistics**		**Pearson's correlations**			
	**Mean**	**SD**	**1**	**2**	**3**	**4**
1. QPR intrapsychic process	2.03	0.83				
2. QPR interpersonal process	2.17	0.89	0.73^**^			
3. FIAQ openness	9.21	4.06	−0.18	−0.16		
4. FIAQ confirmation	7.00	3.39	−0.25	−0.14	0.88^**^	
5. FIAQ cooperation	11.45	5.21	−0.37^*^	−0.26	0.86^**^	0.91^**^

^**^Correlation is significant at the 0.01 level (2-tailed).

^*^Correlation is significant at the 0.05 level (2-tailed).

## Discussion

To our knowledge, this is the first study to involve carers and users of ACT services, and assess personal recovery from their point of view. The results showed that the total sample of caregivers (*n* = 36) reported significantly lower than the total sample of users (*n* = 69) on both the intrapsychic process and the interpersonal subscales of personal recovery. Matching the carers and users (*n* = 18) also showed the same pattern. Our results also showed a significant negative correlation between the carers' experience of cooperation with the ACT teams and their assessment of the intrapsychic recovery process.

### High user rating

Users rated themselves about one standard deviation above the mean on both subscales of personal recovery, placing themselves around or above the 84th percentile. The ACT is a novel approach compared to traditional MHS and expecting change is an important condition that affects the treatment process and outcome. Psychotherapy research shows a connection between expectation and outcome rating (45, 46). However, the lack of pre- and post-measurement in our study limited the scope of the interpretation, and high user rating could be an artifact of the Hawthorne effect, where mere participation in a novel protocol produces better results (47). Furthermore, since carers did not actively participate in the ACT teams, the Hawthorne effect may not have been relevant to them.

### Low carer rating

The low carer rating can be understood in several ways. First, the mean scores of the carers hovered around the middle of a 5-point Likert scale, which means that the informants neither agreed nor disagreed with the statements and remained neutral. However, the neutrality could be a wishful waiting and watching to see more changes over time and in various social/familial contexts. Second, carers' feeling of being excluded by MHS is well documented (5). It is possible that carers' expectancy was already low from the burden of care over a long time-period and poor previous experience with MHS. Therefore, they simply did not find enough reason to expect anything other than low personal recovery of the users. The carers of the ACT users in Norway corroborated this trend by reporting that ACT services were not different from their previous experience with MHS (16). Third, historically, ACT services have been directed toward users and no carer components were built into the model. Although carer involvement is mandated in Norway, they were neither the target group nor a part of the model driving the ACT services (48). Therefore, carers may have been excluded and the consequence was that they rated personal recovery more conservatively than did the users.

### Role of a long-standing relationship

We assumed a long-term, close relationship between carers and users in the matched subgroup (*n* = 18) because carers were parents and siblings. Further, it is reasonable to expect that the matched carer-user subgroup is well acquainted with the idiosyncrasies specific to mental illness and to detect emotional and behavioral changes if present. Based on this assumption, it was interesting to note that intimate knowledge of the user showed a lower carer rating compared to the user. Subscribing to the theory of expressed emotion, the lower carer rating may be a function of being overly critical and overly involved with the user (49). However, since both the total sample and the matched sample in our study had similar results, it suggests that the carers were neutral and waiting to see recovery related benefits over time and across various contexts.

### Association between carers' feeling of involvement and rating of personal recovery

There was no significant association between the carer's experience with involvement and their evaluation of personal recovery, with one exception. There was a significant negative correlation between the FIA-Q cooperation subscale and the QPR intrapsychic process subscale. This was an unexpected result, and we conjecture that the ACT teams may have initiated contact and cooperated with carers to stabilize the user after an adverse illness-related episode. Therefore, carers may have associated high level of cooperation with the ACT team, with low level of the intrapersonal recovery process. It is not clear why only the intrapersonal process scale was associated with cooperation but not the interpersonal scale. There may be a technical explanation. Only 5 items of the QPR make up the interpersonal process sub-scale. Psychometric studies of the QPR found weak internal consistency for the interpersonal subscale and strong support for a single-factor solution consisting of 15 items (50, 51). Therefore, regardless of the direction of the association, the intrapersonal subscale just may have better power to detect the correlation.

### Discrepancy and multi-informant assessments

The discrepancy between informants is an inherent characteristic of multi-informant assessments (15, 16, 51–55). Differing report of the same phenomenon by the carers and users in our study is neither new nor unexpected. There are ways to handle discrepancies in clinical assessments and test if the pattern of convergence and divergence of multi-informant reports can provide meaningful information. The framework of the Operations Triad Model (OTM) (56) proposes that there are expected and meaningful reasons for the divergence. For example, expecting divergent information on how the underlying pathology manifests in a different context (home vs. hospital) is intuitive and meaningful. Such expected differences enrich the information base and contribute toward better clinical decisions. However, divergence can occur due to measurement errors or methodological shortcomings, such as using measures with different content and psychometric properties. Differentiating between the two sources and critically assessing the information provides a way to expand the information base, minimize systematic errors in handling multi-informant assessments, and provide a rich information base to make clinical decisions.

However, it may be challenging to secure these strategies in the clinic, given that clinicians may prefer a simple one-source account from the user/patient. A recent experimental study highlighted the complexity of dealing with discrepant reports in MHS. Marsh et al. (57) conducted four experimental studies and showed that when faced with discrepant information, laypeople and MHS professionals choose to trust one of the informants. Additionally, the choice of whether to trust the client or the informant is based on the specific mental health condition (internalizing or externalizing), the relationship with the client (close or distant), and the nature of the report (optimism vs. pessimism). Carers close to the user who were pessimistic about internalizing conditions were deemed knowledgeable and credible. Likewise, carers who were distant and pessimistic informants of externalizing conditions were deemed more knowledgeable and credible. The information from this study informs how the various conditions could affect the caregiver's report, how they could be evaluated, and how they can be gainfully incorporated into MHS.

### Clinical implications

The discussion above gives reason for optimism about including carers in assessing personal recovery and other clinical processes. The carers were neutral in their evaluation of personal recovery. Given the lack of experience with ACT services, one would expect neutrality. Therefore, we propose that carer rating would provide valuable third-party information beyond MHS context. Clinicians can be skeptical about various sources of information sources, especially if it is conflicting information. However, the framework of OTM (56) and the experimental study of Marsh et al. (57) showed that simply being a conservative or pessimistic informant does not disqualify or discredit carers. There are conditions and criteria for which conservative informants are considered knowledgeable and credible compared to caregivers who are optimistic informants. Listening to carers' voices and carefully interpreting divergence/convergence of information provides a theoretical and methodological foundation to involve carers in planning, tailoring, and evaluating, ultimately benefiting the users.

## Limitations and future research

The strengths of our study were the use of the clinical sample and the conduct of the first study examining the participation of carers in ACT services in Norway. However, our study also has some limitations. First, to our knowledge, our study is the first to have modified the QPR for use with carers. Therefore, our findings rest on the notion that this approach is an acceptable way for carers to assess personal recovery. Since the alpha values for the modified QPR were high, we are optimistic. A related matter is that the present version of QPR may not have fully captured the interpersonal and relational aspects of personal recovery. Furthermore, our understanding of user reactions to carer evaluation of personal recovery is limited. Qualitative research may be necessary to obtain a more comprehensive understanding of this phenomenon, and future investigations may want to consider this approach. Third, ACT teams in Norway used the “Tool for Measurement of Assertive Community Treatment (TMACT) version 1. Therefore, our results should be understood in the context where there was a lack of systematic inclusion of carers and evaluation of family focused interventions in ACT teams, and generally in the broader clinical setting. Lastly, our study's limited number of participants and use of convenience sampling constrained the scope of research questions, statistical analyses, and generalizability. Future studies with larger sample sizes, particularly of matched pairs, together with a larger battery of measures and covariates, and a qualitative section, would provide more conclusive results and help critical appraisal of the familial and relational aspects of personal recovery.

## Conclusions

Including the carers in the assessment of ACT services and the results when compared to the users, is in line with previous clinical literature regarding MHS to individuals with SMI. Discrepancy is an essential feature of the multi-informant methodology. As our study showed, understanding how informants differ and the conditions that affect the direction of the assessments provide a rationale for clinicians and stakeholders in MHS to involve the carers in the assessment and treatment of individuals with SMI. The proactive involvement of the carers would provide valuable third-party information, aid in the treatment process, and help improve the quality of the ACT teams.

## Data availability statement

The raw data supporting the conclusions of this article will be made available by the authors, without undue reservation.

## Ethics statement

The studies involving human participants were reviewed and approved by Southeast Regional Ethics Committee for Medical and Health Research (Reg. No. 2010/1196a) and the data protection officer at Akershus University Hospital (Reg. No. 2012/094). The patients/participants provided their written informed consent to participate in this study.

## Author contributions

PI wrote the first draft of the manuscript. All authors contributed to the conception and design of the study, data collection, contributed to the manuscript revision, and read and approved the submitted version.
